# Efficacy of breast reconstruction for N2‐3M0 stage female breast cancer on breast cancer‐specific survival: A population‐based propensity score analysis

**DOI:** 10.1002/cam4.6579

**Published:** 2023-10-05

**Authors:** Yuting Zhao, Lutong Yan, Shouyu Li, Zejian Yang, Na Chai, Pei Qiu, Huimin Zhang, Jianjun He, Can Zhou

**Affiliations:** ^1^ Department of Gynecologic Oncology National Cancer Center/National Clinical Research Center for Cancer/Cancer Hospital, Chinese Academy of Medical Sciences and Peking Union Medical College Beijing China; ^2^ Chinese Academy of Medical Sciences & Peking Union Medical College Beijing China; ^3^ Department of Pediatric surgery The First Affiliated Hospital of Xi'an Jiaotong University Xi'an China; ^4^ Department of Breast Surgery The First Affiliated Hospital of Xi'an Jiaotong University Xi'an China; ^5^ School of Medicine Xi'an Jiaotong University Xi'an China

**Keywords:** breast cancer, breast cancer‐specific survival, breast reconstruction, generalized boosted model, propensity score matching, SEER

## Abstract

**Background:**

The efficacy of breast reconstruction for patients with N2‐3M0 stage female breast cancer (FBC) remained unclear due to the lack of randomized clinical trials. This retrospective study aimed to explore the efficacy of breast reconstruction for patients with N2‐3M0 stage FBC.

**Methods:**

Two thousand five hundred forty‐five subjects with FBC staged by N2‐3M0 from 2010 to 2016 were retrieved from the Surveillance, Epidemiology, and End Results database. Generalized boosted model (GBM) and propensity score matching (PSM) analyses and multivariable Cox analyses were employed to assess the clinical prognostic effect of postmastectomy reconstruction for patients with N2‐3M0 stage FBC in breast cancer‐specific survival (BCSS).

**Results:**

Totally, 1784 candidates underwent mastectomy alone (mastectomy group), and 761 candidates underwent postmastectomy reconstruction (PMbR group), with 418 breast‐specific deaths after a median follow‐up time of 57 months (ranging from 7 to 227 months). BCSS in the mastectomy group showed no statistical difference from that in the PMbR group in the PSM cohort (HR = 0.93, 95% CI: 0.70–1.25, *p* = 0.400) and GBM cohort (HR = 0.75, 95% CI: 0.56–1.01, *p* = 0.057). In the multivariate analyses, there was no difference in the effect of PMbR and mastectomy on BCSS in the original cohort (HR = 0.85, 95% CI: 0.66–1.09, *p* = 0.197), PSM cohort (HR = 0.86, 95% CI: 0.64–1.15, *p* = 0.310), and GBM cohort (HR = 0.84, 95% CI: 0.61–1.17, *p* = 0.298). Triple‐negative breast cancer (TNBC) was a detrimental factor affecting BCSS for patients in the PMbR group.

**Conclusions:**

Our study demonstrated that PMbR is an oncologically safe surgical treatment and can be widely recommended in clinics for females with non‐TNBC staged by T0‐3N2‐3M0.

## 1 INTRODUCTION

Postmastectomy breast reconstruction (PMbR) is designed to help female patients who underwent a mastectomy to reshape the appearance and anatomical landmarks of the breast, restore the integrity of the body shape, and achieve as much symmetry as possible on both sides of the breast, has evolved a gradual rise over the past few decades.^1^, ^2^, ^3^ What's more, research proved PMbR to be oncologically safe and not affect long‐term survival.^4^, ^5^, ^6^ PMbR means the maintenance of the well‐being of the female by keeping the shape of the breast and improved esthetic outcomes and satisfaction without reducing oncologic safety in the survival or early diagnosis of local failure of breast cancer.^7^, ^8^ And PMbR has been recommended as the principal choice of treatment for patients with mammectomy by the national comprehensive cancer network (NCCN) guidelines and the European Society for Medical Oncology Clinical Practice Guidelines (ESMO).^9^, ^10^

The long‐term oncologic safety and feasibility are supported by a succession of studies in prestigious journals.^11^, ^12^, ^13^, ^14^ And the paradigmatic evolution in long‐term oncologic outcomes and demonstrations of its oncologic safety profile help and encourage the doctors and patients to accept, popularize, and refine the rising awareness of PMbR for FBC. The proportion of the modus operandi is reported to have increased from 1995 (8%) to 2013 (41%).^15^, ^16^ However, the proportion of N0‐1 stage female breast cancer (FBC) patients performed with PMbR is more than 80% in real‐world clinical work^17^ and between 74.4% and 96.8% in most clinical trials.^11^, ^13^, ^14^ These could be attributed to the lack of fully appreciated oncologic safety and feasibility of breast reconstruction for patients with N2‐3M0 stage FBC due to the loss of specific real‐world clinical work or randomized controlled trials (RCTs).^18^ And therefore, the real‐world effectiveness of PMbR on the survival of females with N2‐3M0 stage breast cancer deserves further exploration and discussion.

To explore the treatment outcome of PMbR on FBC patients with N2‐3M0 stage, subjects of postoperative patients with FBC derived from the Surveillance, Epidemiology, and End Results (SEER) database were analyzed with the generalized boosted model (GBM) and propensity score matching (PSM) analyses.

## 2 MATERIALS AND METHODS

### 2.1 Data sources, patients, and variables selection

Data for this study were generated with the SEER*Stat program (version 8.3.8, http://seer.cancer.gov/seerstat, Information Management Service, Inc.). Patients with FBC diagnosed from 2010 to 2016 were recruited with the criteria as follows: (1) primary tumor diagnosis of breast cancer; (2) Breast‐Adjusted American Joint Committee on Cancer (AJCC) 6th N2‐3 and M0 stage; (3) cancer‐directed surgery recode mastectomy with or without reconstruction.

**FIGURE 1:**
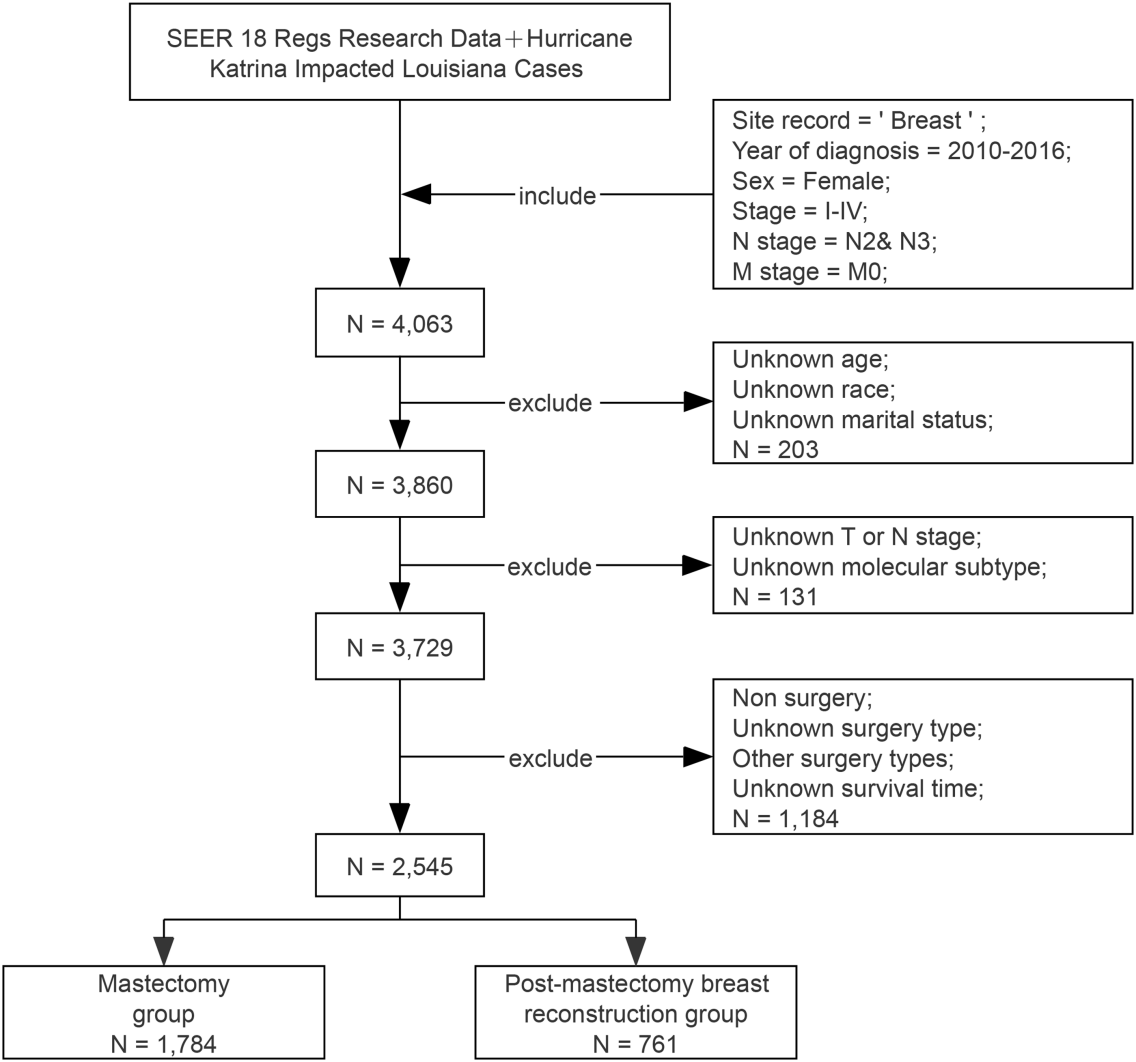
Flowchart of the study design. SEER, the Surveillance, Epidemiology, and End Results database.

Subjects meeting the following criteria were excluded: (1) unknown age at diagnosis, marital status, AJCC stage, survival time, estrogen receptor (ER) status, progesterone receptor (PR) status, or human epidermal growth factor receptor 2 (HER2) status; (2) the diagnosis methods of autopsy only or death certificate; (3) AJCC stage IV or N0‐1 and M0 stage; (4) missing surgical records or surgery not performed. Figure 1 illustrated the study design and the filtering procedure. In total, 2545 patients were recruited in the current research, including 1784 patients who underwent conventional mastectomy (mastectomy group) and 761 patients who underwent PMbR (PMbR group).

Subjects were followed up with a median follow‐up time of 3.50 years (range, 0 months to 6.92 years). The endpoint of this study was breast cancer‐specific survival (BCSS), defined as the time from the date of diagnosis until death related to breast cancer.

### 2.2 Statistical analysis

PSM and GBM analyses were performed to balance the baseline features between mastectomy and PMbR groups. Logistic regression analysis was established to calculate propensity scores by study variables. In the PSM analysis, the clinicopathological parameters were matched according to propensity scores with the ratio of 1:1 through the nearest neighbor matching with the caliper of 0.2 in mastectomy and PMbR groups.

Observations were weighted by the inverse of the calculated probability of receiving the observed sequence of treatments the individual received, referred to as an inverse probability of treatment weight (IPTW) analysis. As a machine‐learning method of implementing IPTW and based on a decision‐tree model, the GBM analysis could create complex models by combining multiple simple models through an iterative algorithm to produce a better predictive performance than any simple model, with no loss of sample size.^18^ After estimating the relevant propensity scores and testing the model, we translated the propensity score ($ê\;\left(x\right)$) into analytic weights and assessed the treatment effects of PMbR. The weight of the average treatment effect (ATE) was estimated as follows:

For objects in the PMbR group:

$$\omega =\frac{1}{ê\;\left(x\right)}$$

For objects in the mastectomy group:

$$\omega =\frac{1}{1-ê\;\left(x\right)}$$

Each research object was given a corresponding weight for weighting through the propensity score value by the principle of the standardization method. Then, the distribution of the propensity score and confounding factors was consistent in each group. Pearson's chi‐square test, Fisher's exact test, and standardized mean difference (SMD) were applied to compare the differences between mastectomy and PMbR groups. SMD less than 0.1 indicated an excellent balance effect.

Hazard ratios (HR) with 95% confidence intervals (CIs) were calculated by the Cox proportional hazards model to estimate risk factors affecting BCSS. The Cox proportional hazards models were tested by the likelihood ratio and Wald test. Survival curves were plotted through the Kaplan–Meier analyses. Two‐sided *p* < 0.05 was considered to have statistical significance. Statistical analyses were conducted in software package R version 4.1.2. GBM was fitted using the *TWANG* R package (https://www.rand.org/statistics/twang.html).

## 3 RESULTS

### 3.1 Patient characteristics

**FIGURE 2:**
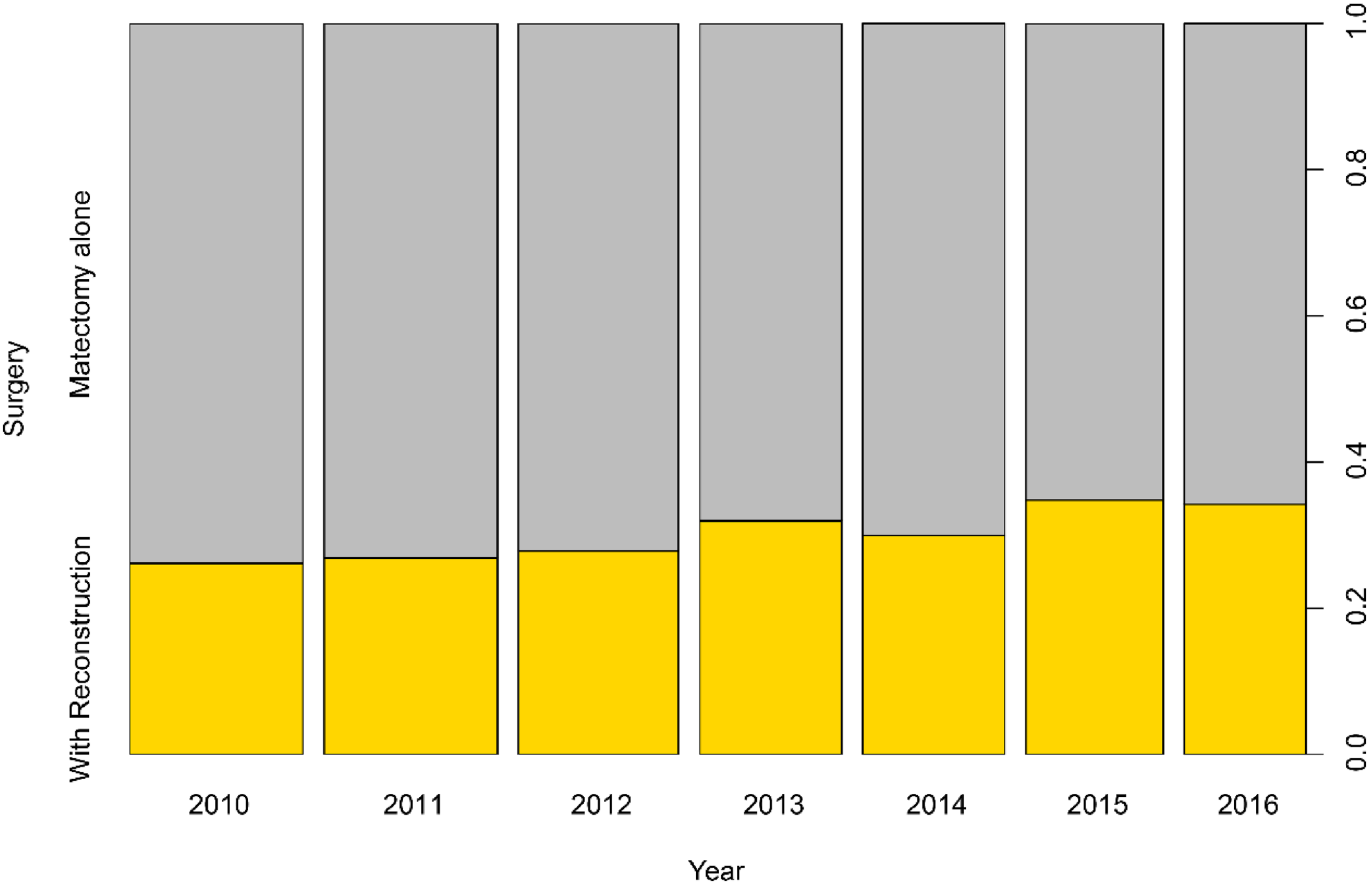
The rate of patients received mastectomy only and mastectomy with reconstruction.

Among the 2545 subjects recruited in this study, a total of 761 (30.0%) patients had undergone postmastectomy breast reconstruction (PMbR group), and 1784 (70.0%) patients had undergone mastectomy only (mastectomy group). The rate of PMbR increased from 26.1% in 2010 to 34.2% in 2016, with an absolute increase of 6.1% (Figure 2). Significant differences in baseline features were observed between the PMbR and mastectomy groups (Table 1) in the original cohort. PSM and GBM analyses were performed to avoid or diminish the potential prognostic confounding biases. The differences in the clinicopathological features between PMbR and mastectomy groups vanished or weakened in both the PSM and GBM cohorts. The establishment procedure of the GBM model was shown in Figure S1, that each panel of the optimize plot (Figure S1A) indicated the minimized stopping rule in the GBM model, plots of *p*‐value (Figure S1B), as well as effect size plots (Figure S1C), indicating the excellent balance effect of the GBM model.

**TABLE 1:** Clinicopathological characteristics of the mastectomy and PMbR groups in the original cohort, matched cohort and weighted cohort.

Covariates	Original Cohort				Matched Cohort				Weighted Cohort			
	Mastectomy (%) *n* = 1784	PMbR (%) *n* = 761	*p* *	SMD	Mastectomy (%) *n* = 712	PMbR (%) *n* = 712	*p* *	SMD	Mastectomy (%) *n* = 2534	PMbR (%) *n* = 2258	*p* *	SMD
Age at diagnosis												
<40	120 (6.7)	134 (17.6)	<0.001	0.717	100 (14.0)	113 (15.9)	0.579	0.055	248 (9.8)	247 (11.0)	0.076	0.125
40–64	933 (52.3)	542 (71.2)			530 (74.4)	514 (72.2)			1473 (58.1)	1413 (62.6)		
≥65	731 (41.0)	85 (11.2)			82 (11.5)	85 (11.9)			814 (32.1)	597 (26.4)		
Race												
White	1176 (65.9)	593 (77.9)	<0.001	0.285	540 (75.8)	545 (76.5)	0.945	0.018	1766 (69.7)	1632 (72.3)	0.486	0.066
Black	333 (18.7)	107 (14.1)			108 (15.2)	106 (14.9)			435 (17.2)	372 (16.5)		
Others	275 (15.4)	61 (8.0)			64 (9.0)	61 (8.6)			333 (13.1)	253 (11.2)		
Marital status												
Married	893 (50.1)	497 (65.3)	<0.001	0.312	447 (62.8)	449 (63.1)	0.956	0.006	1385 (54.7)	1280 (56.7)	0.441	0.041
Unmarried/Loss of marriage	891 (49.9)	264 (34.7)			265 (37.2)	263 (36.9)			1149 (45.3)	978 (43.3)		
Grade												
I	160 (9.0)	82 (10.8)	<0.001	0.190	73 (10.3)	72 (10.1)	0.964	0.014	241 (9.5)	246 (10.9)	0.498	0.063
II	745 (41.8)	384 (50.5)			355 (49.9)	351 (49.3)			1127 (44.5)	1033 (45.7)		
III–IV	879 (49.3)	295 (38.8)			284 (39.9)	289 (40.6)			1167 (46)	979 (43.4)		
T stage												
0–1	236 (13.2)	128 (16.8)	<0.001	0.274	122 (17.1)	118 (16.6)	0.900	0.041	365 (14.4)	367 (16.3)	0.095	0.142
2	759 (42.5)	358 (47.0)			349 (49.0)	340 (47.8)			1112 (43.9)	1012 (44.8)		
3	495 (27.7)	239 (31.4)			205 (28.8)	218 (30.6)			729 (28.8)	682 (30.2)		
4	294 (16.5)	36 (4.7)			36 (5.1)	36 (5.1)			329 (13.0)	196 (8.7)		
N stage												
2	1099 (61.6)	513 (67.4)	0.005	0.122	475 (66.7)	467 (65.6)	0.695	0.024	1604.0 (63.3)	1445 (64.0)	0.793	0.014
3	685 (38.4)	248 (32.6)			237 (33.3)	245 (34.4)			930 (36.7)	813 (36.0)		
Molecular subtype												
Hormone receptor+/HER 2−	1124 (63.0)	555 (72.9)	<0.001	0.222	513 (72.1)	511 (71.8)	0.954	0.031	1675 (66.1)	1567 (69.4)	0.541	0.076
Hormone receptor+/HER 2+	258 (14.5)	93 (12.2)			84 (11.8)	89 (12.5)			348 (13.7)	294 (13)		
Hormone receptor−/HER 2+	143 (8.0)	43 (5.7)			47 (6.6)	43 (6.0)			184 (7.3)	135 (6)		
Hormone receptor−/HER 2−	259 (14.5)	70 (9.2)			68 (9.6)	69 (9.7)			327 (12.9)	262 (11.6)		
Radiotherapy												
No/unknown	573 (32.1)	215 (28.3)	0.053	0.084	188 (26.4)	207 (29.1)	0.287	0.060	779 (30.8)	671 (29.7)	0.665	0.023
Yes	1211 (67.9)	546 (71.7)			524 (73.6)	505 (70.9)			1755 (69.2)	1587 (70.3)		
Chemotherapy												
No/unknown	380 (21.3)	52 (6.8)	<0.001	0.425	49 (6.9)	52 (7.3)	0.836	0.016	430 (17.0)	286 (12.6)	0.082	0.123
Yes	1404 (78.7)	709 (93.2)			663 (93.1)	660 (92.7)			2104 (83.0)	1972 (87.4)		

Abbreviation: HER2, human epidermal growth factor receptor 2; PMbR, postmastectomy breast reconstruction SMD, standardized mean difference.

* *p*‐values showed whether there were statistical differences among, new *p*‐values shall be shown, when necessary, in the below.

### 3.2 The Effect of postmastectomy reconstruction on breast cancer‐specific survival for patients with N2‐3 stage breast cancer

**FIGURE 3:**
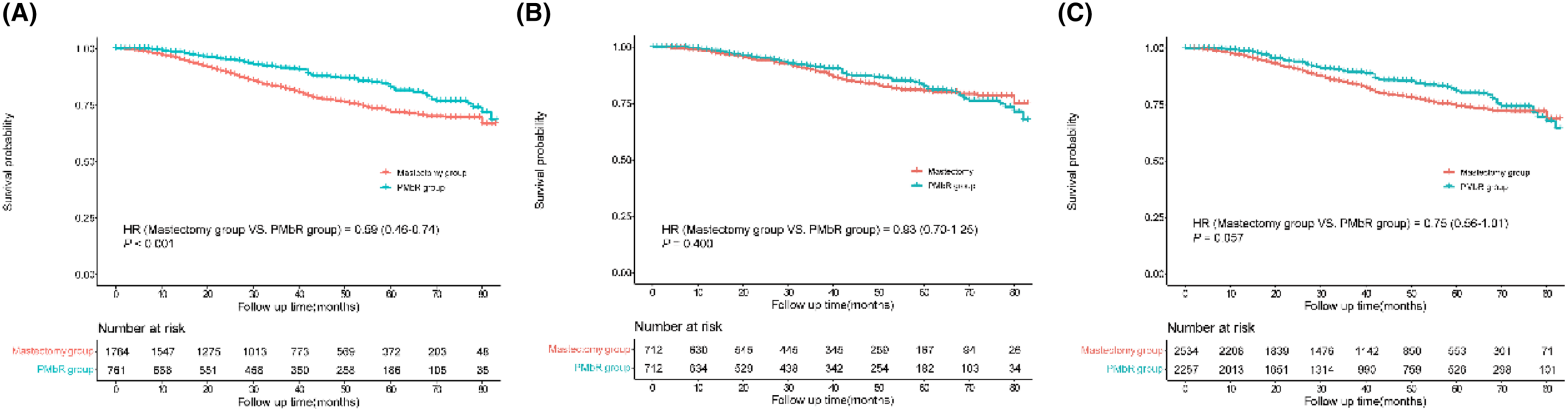
Comparison of breast cancer‐specific survival between mastectomy group and PMbR group. Kaplan–Meier analysis and log‐rank test in the original cohort (A), matched cohort (B), and weighted cohort (C). HR, hazard ratio; PMbR, postmastectomy breast reconstruction.

Subjects of the PMbR group tended to show more favorable BCSS in all the original cohort (HR = 0.59, 95% CI: 0.46–0.74, *p* < 0.001; Figure 3A) than those in the mastectomy group. However, BCSS in the mastectomy group showed no statistical difference from that in the PMbR group in the PSM cohort (HR = 0.93, 95% CI: 0.70–1.25, *p* = 0.400) and GBM cohort (HR = 0.75, 95% CI: 0.56–1.01, *p* = 0.057). The cumulative incidence of 5‐year BCSS in the PMbR and mastectomy groups was 82.5% and 78.1% in the PSM cohort, 81.0% and 74.1% in the GBM cohort, and 79.2% and 72.0% in the original cohort, respectively.

### 3.3 Multivariate Cox regression analysis on the factors affecting breast cancer‐specific survival

**FIGURE 4:**
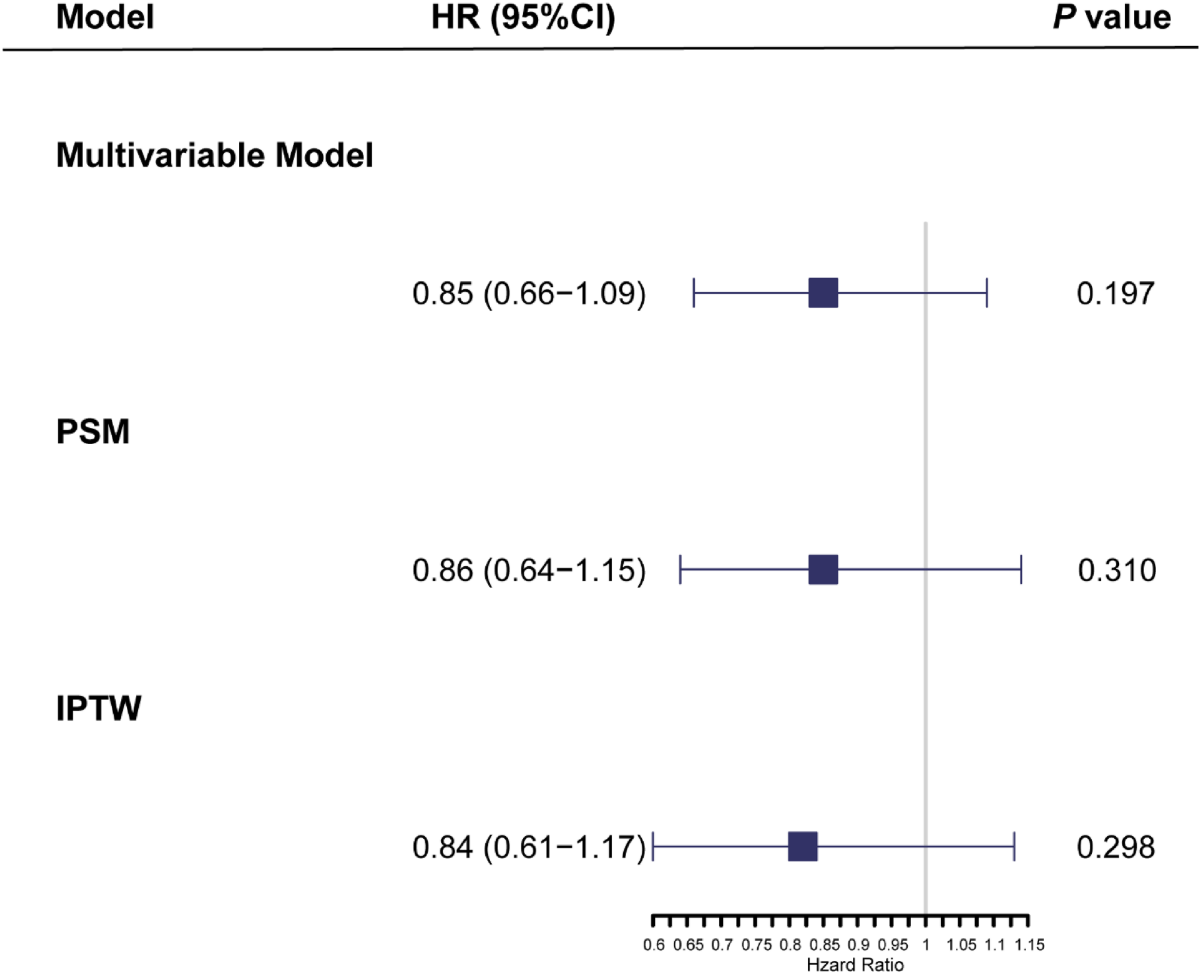
Hazard ratios of PMbR compared with mastectomy in the multivariable Cox model, propensity score matching model, and generalized boosted model. CI, confidence interval; IPTW, inverse probability of treatment weight; PMbR, postmastectomy breast reconstruction; PSM, propensity score matching.

To ensure more robust results, the multivariate Cox analysis was conducted in the PSM, GBM, and original cohorts (Table S1). There was no statistical difference in the effect of PMbR versus mastectomy on BCSS in the PSM cohort (HR = 0.86, 95% CI: 0.64–1.15, *p* = 0.310), GBM cohort (HR = 0.84, 95% CI: 0.61–1.17, *p* = 0.298), and original cohort (HR = 0.85, 95% CI: 0.66–1.09, *p* = 0.197) (Figure 4). In addition, some adverse predictors of BCSS, such as grade III–IV (*p* < 0.05), T4 stage (*p* < 0.05), N3 stage (*p* < 0.05), triple‐negative subtype (*p* < 0.001), and two beneficial predictors of BCSS, radiotherapy (*p* < 0.05) and chemotherapy (*p* < 0.05), were revealed in the Cox analyses in all the PSM, GBM, and original cohorts.

### 3.4 Multivariate Cox regression analysis on the factors affecting breast cancer‐specific survival for patients in the postmastectomy reconstruction group

**FIGURE 5:**
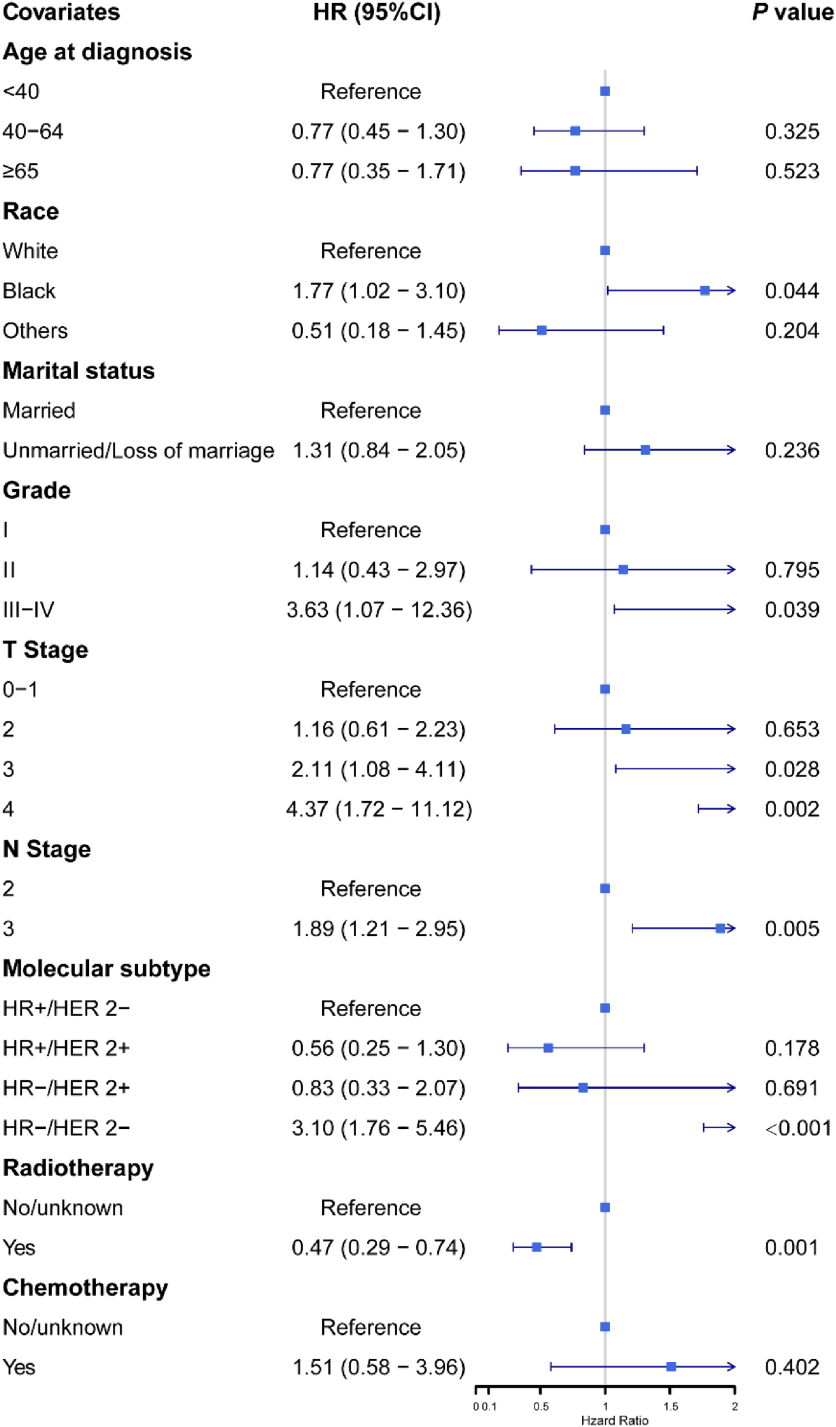
Forest plot for multivariate Cox analysis of breast cancer‐specific survival predictors in the PMbR group. CI, confidence interval; HER2, human epidermal growth factor receptor 2; PMbR, postmastectomy breast reconstruction.

To further estimate the unfavorable factors of PMbR, a multivariate Cox analysis was conducted in the PMbR group of the original cohort. As shown in Figure 5, histopathological grade of III–IV levels (HR = 3.63, 95% CI: 1.07–12.36, *p* = 0.039), T4 stage (HR = 4.37, 95% CI: 1.72–11.12, *p* = 0.002), N3 stage (HR = 1.89, 95% CI: 1.21–2.95, *p* = 0.005), and triple‐negative subtype (HR = 3.10, 95% CI: 1.76–5.46, *p* < 0.001) were still detrimental factors of BCSS. In addition, radiotherapy (HR = 0.47, 95% CI: 0.29–0.74, *p* = 0.001) was a protective factor of prognosis for patients who underwent mastectomy with reconstruction.

### 3.5 The effect of histopathological grade level, T stage, N stage, and molecular subtype on breast cancer‐specific survival for patients in the postmastectomy reconstruction group

**FIGURE 6:**
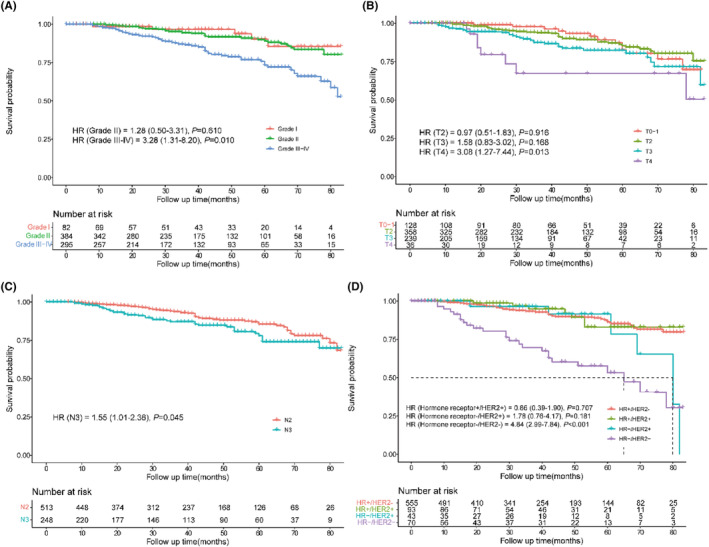
Subgroup analyses stratified (A) by grade, (B) by T stage, (C) by N stage, and (D) by molecular subtype. *p*‐value was calculated by log‐rank test. HER2, human epidermal growth factor receptor 2; HR, hazard ratio.

Survival analyses were performed to explore the impact of histopathological grade, T stage, N stage, and molecular subtype on BCSS of patients in the PMbR group. As shown in Figure 6, grade level of III–IV (HR = 3.28, 95% CI: 1.31–8.20, *p* = 0.010), T4 stage (HR = 3.08, 95% CI: 1.27–7.44, *p* = 0.013), N3 stage (HR = 1.55, 95% CI: 1.01–2.36, *p* = 0.045), and triple‐negative breast cancer (TNBC) (HR = 4.84, 95% CI: 2.99–7.84, *p* < 0.001) were unfavorable factors of BCSS for FBC patients with N2‐3 stage. The cumulative incidence of 5‐year BCSS was 73.3% in the grade III–IV subgroup, 67.3% in the T4 stage subgroup, 78.1% in the N3 subgroup, and 53.2% in the TNBC subgroup.

### 3.6 Sensitivity analysis

In order to examine whether there were other potential confounding factors not included in the PS calculation, sensitivity analysis was conducted by including three variables: tumor laterality, histological type, and the number of resected lymph nodes (LN). Table S2 showed the distribution of these three variables in the original cohort, indicating no differences between the two groups. PS calculations were performed for PSM and GBM in the sensitivity analysis (Table S2), respectively. After including the three variables, Cox analyses were conducted in the original cohort, PSM cohort, and GBM cohort, respectively. Table S3 showed that tumor laterality (Right, original cohort: HR = 1.09, *p* = 0.377; PSM cohort: HR = 1.25, *p* = 0.169; GBM cohort: HR = 1.18, *p* = 0.176), histological type (ILC, original cohort: HR = 1.05, *p* = 0.768; PSM cohort: HR = 1.12, *p* = 0.658; GBM cohort: HR = 1.04, *p* = 0.850; Others, original cohort: HR = 0.98, *p* = 0.952; PSM cohort: HR = 1.06, *p* = 0.904; GBM cohort: HR = 1.39, *p* = 0.416), and LN examined (>15, original cohort: HR = 0.83, *p* = 0.075; PSM cohort: HR = 0.82, *p* = 0.213; GBM cohort: HR = 0.89, *p* = 0.381) had no independent impact on BCSS. In the sensitivity analysis, the HR of PMbR for BCSS was similar in the original cohort (HR = 0.62, 95% CI: 0.46–1.02, *p* = 0.131), PSM cohort (HR = 0.67, 95% CI: 0.49–1.02, *p* = 0.115), and GBM cohort (HR = 0.62, 95% CI: 0.54–1.08, *p* = 0.137) compared to the previous analysis.

## 4 DISCUSSION

Owing to the deepening regarding of FBC, rapid development in oncological therapeutic areas, and establishment of its oncological safety profile in breast cancer treatment, the acquaintances and innovations in theories and reconstructive techniques of PMbR have evolved in recent years.^1^, ^2^, ^3^, ^19^, ^20^ Consistent with previous reports,^19^, ^20^ in the current study, we found that the ratio of patients who received PMbR rose from 2010 (26.1%) to 2016 (34.2%). Such results indicated that a growing number of people with breast cancer tended to accept the reconstructive technique of PMbR to maintain a high quality of life and body image.^21^, ^22^

As previously mentioned, the reconstructive technique of PMbR has been recommended to be eligible for all FBC patients with mammectomy by NCCN and ESMO guidelines.^9^, ^10^ However, the effect and safety of PMbR remained controversial for patients with N2‐3M0 stage FBC. On the one side, PMbR means restoring the mound of breast and improving esthetic outcomes.^7^, ^8^ On the other side, the N2‐3M0 stage means a high risk of FBC recurrence and metastasis, poor long‐term prognosis, and reluctance to accept the reconstructive technique of PMbR.^8^, ^11^ Therefore, it was of necessity to investigate the efficiency and safety of PMbR for patients staged by N2‐3M0 FBC undergoing PMbR. This study investigated the relationship between PMbR and BCSS for patients with N2‐3 stage breast cancer among the large cohort of 2545 subjects after surgery. It was found in this study that patients in the PMbR group showed similar BCSS with those in the mastectomy. As far as we know, it was the first large‐cohort research to investigate the effect of PMbR on BCSS for FBC patients staged by N2‐3M0 through PSM and GBM methods and survival analyses.

Clinical and pathological characteristics, such as age, histologic grade, marital status, stage, and so on, have always been regarded as essential criteria that help to establish reasonable clinical protocols in patients with FBC. This research revealed significant differences in the clinicopathological characteristics, such as age, married status, and chemotherapy status between the PMbR and mastectomy groups. To balance the differences between groups and supplement the lack of RCTs, both PSM and GBM analyses were performed. As a novel method of propensity scoring applied to medical research, the performance of GBM analysis played a vital role in balancing the clinicopathological features. It could overcome the disadvantage of wasting the sample size by PSM analysis, which was often used to make characteristics between groups balanced and comparable in observational studies.^23^ Expectedly, nearly no differences in the clinicopathological features remained in the current research. In the Kaplan–Meier survival analyses, patients in the PMbR had a similar BCSS to those in the mastectomy group after PSM and GBM analyses, indicating the safety of PMbR in patients with N2‐3 stage. However, there existed potential bias suffering from unbalanced confounding factors affecting the results. The patients in the PMbR group tended to have higher income, private health insurance, better access to health care, and fewer comorbidities,^24^, ^25^ which we could not access and balance using either PSM or GBM.

Kaplan–Meier analyses and multivariate Cox models are utilized to further estimate the effectiveness of PMbR for subjects with N2‐3M0 stage FBC. According to our survival analyses, the reconstructive technique of PMbR is safe for patients with N2‐3M0 stage FBC in comparison with those with mastectomy. This finding coincides with those reported in the literature that the reconstructive technique of PMbR is oncologically safe for patients with advanced nodal stage FBC.^26^, ^27^, ^28^, ^29^, ^30^ The existence of the mound of breast means more psychosocial and improved esthetic outcomes, and PMbR provided the benefit in terms of survival and esthetics for female patients with N2‐3 disease. Therefore, the reconstruction technique would attain a similar BCSS as mastectomy and could be one of the eligible options of surgery for FBC patients staged by N2‐3M0. And the identification of appropriate patients suitable for PMbR surgery is of great significance.

To further estimate the factors affecting BCSS and identify the patient suitable for PMbR surgery, a multivariate Cox analysis is performed on the clinicopathological features of patients in the PMbR group. In our study, grade III–IV in the degree of cell differentiation, T4 stage, and triple‐negative subtype FBC are adversely independent factors affecting BCSS for patients with N2‐3M0 stage FBC. Such results demonstrated that the assessment of appropriate patients suitable for PMbR should be carefully made. The surgery of PMbR could not be chosen for patients with T4N2‐3M0 stage or N2‐3M0 stage triple‐negative breast cancer as the standard treatment because they provide decreased BCSS. And PMbR could be recommended for females with non‐triple‐negative FBC staged by T0‐3N2‐3M0.

There were some limitations in this study, so conclusions should be explained carefully. First, the retrospective design would suffer from potential biases, including selection bias, confounding bias, and so on. Second, potential confounding biases led by unmeasured factors, which were not available in the SEER database or we could not access, such as insurance status, socioeconomic status, education level, BMI, comorbidity, smoking status, etc.^31^, ^32^ could not be controlled by PSM and GBM. However, patients in the PMbR group tended to be higher income, better educated, and better insured, which could cause potential bias. Third, data about recurrence, complications, as well as some breast cancer‐related biomarkers were unavailable for us to investigate. Recurrence is an important endpoint for assessing the prognosis of patients with N2‐3 stage, but this study failed to estimate the impact of PMbR on recurrence rates. In addition, due to the nature of the algorithm of propensity scoring, the forced balance of covariates might aggravate the imbalance of the distribution of unmeasured variables, thus aggravating the bias in treatment effect estimation.^33^

## 5 CONCLUSIONS

In conclusion, this study found an increasing tendency for modern female patients with breast cancer of advanced nodal stage to choose breast reconstruction. And the reconstructive technique of postmastectomy reconstruction is a safe and efficient option of operative therapy and could be recommended for non‐triple‐negative FBC patients staged by T0‐3N2‐3M0.

## AUTHOR CONTRIBUTIONS

**Yuting Zhao:** Conceptualization (equal); formal analysis (equal); methodology (lead); software (lead); writing – original draft (lead). **Lutong Yan:** Formal analysis (equal); methodology (equal); writing – original draft (equal). **Shouyu Li:** Data curation (lead); methodology (equal); visualization (lead). **Zejian Yang:** Software (equal); validation (equal). **Na Chai:** Investigation (equal); software (equal). **pei qiu:** Investigation (equal). **Huimin Zhang:** Resources (lead). **Jianjun He:** Project administration (lead); supervision (lead); writing – review and editing (equal). **Can Zhou:** Conceptualization (equal); funding acquisition (lead); writing – review and editing (lead).

## CONFLICT OF INTEREST STATEMENT

The authors declare that they have no competing interests.

## ETHICS STATEMENT

This study was approved by the Ethical Committee of the First Affiliated Hospital of Xi'an Jiaotong University. The SEER data erase the identity information of patients, so there is no need for informed consent from the patients.

## Supporting information


Supplementary Information

## Data Availability

The datasets analyzed during the current study are available in the SEER Program (www.seer.cancer.gov) SEER*Stat Database: Incidence – SEER 18 Regs Research Data + Hurricane Katrina Impacted Louisiana Cases, Nov 2017 Sub (1973‐2015 varying).
