# Supplementary material for: Efficacy of breast reconstruction for N2‐3M0 stage female breast cancer on breast cancer‐specific survival: A population‐based propensity score analysis
Source: Cancer Med. 2023 Oct 5;12(20):20287–98. doi: 10.1002/cam4.6579 (PMC10652306; doi:10.1002/cam4.6579)
Supplement: Supplementary file 1 — Supplementary Information [file CAM4-12-20287-s001.pdf]

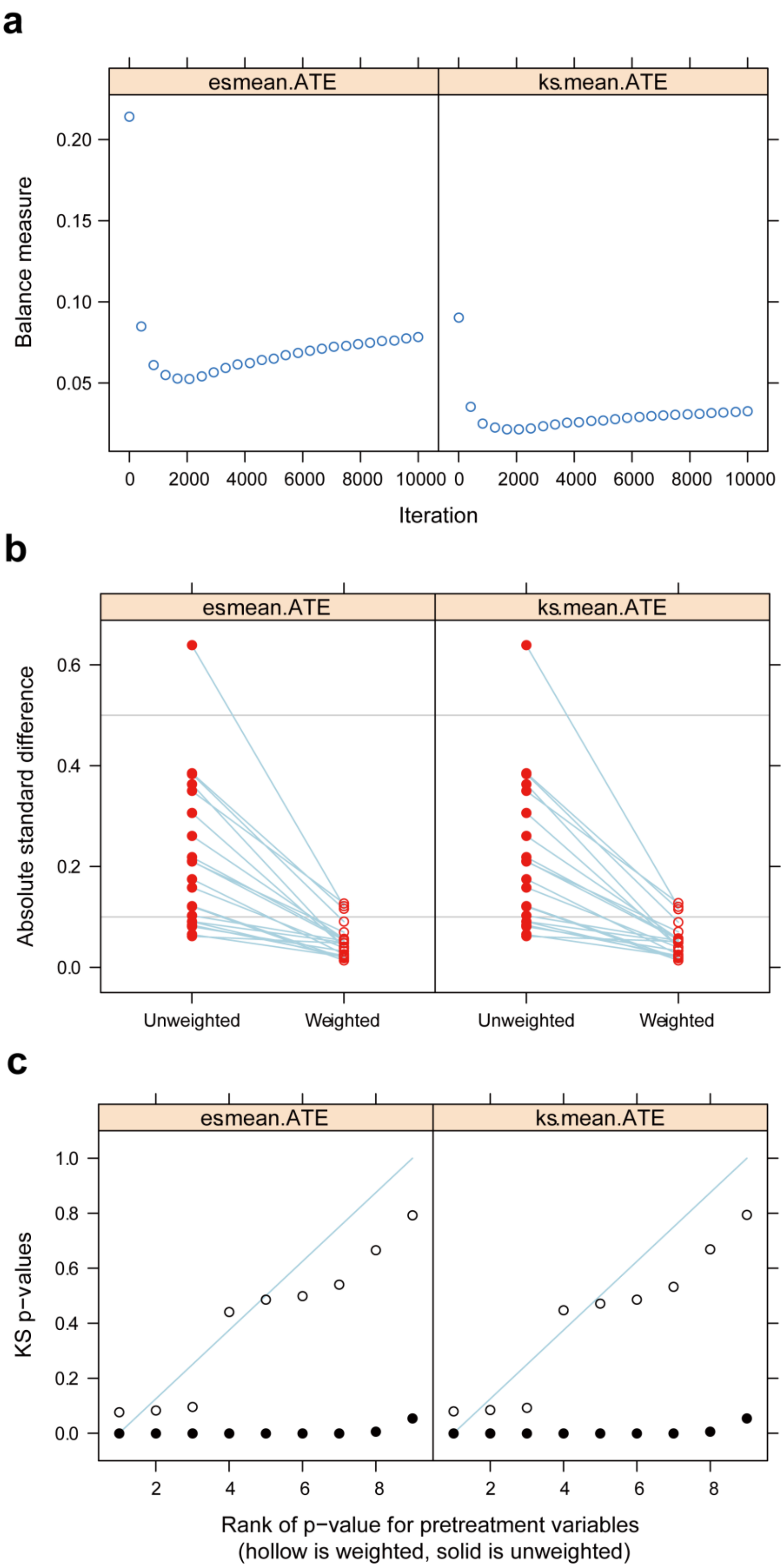

Figure S1. The optimize plot (a), plots of *P* value (b), and effect size plots (c) of the generalized boosted model.

Table S1. Multivariate analysis of breast cancer-specific survival predictors in the original cohort using Cox proportional hazard model.

Covariates	Original Cohort			Matched Cohort			Weighted Cohort		
	HR	*P**	95% CI	HR	*P**	95% CI	HR	*P**	95% CI
**Age at diagnosis**									
<40	Reference			Reference			Reference		
40-64	0.71	0.031	0.51-0.97	0.67	0.030	0.46-0.96	0.72	0.101	0.49-1.07
≥65	0.91	0.603	0.64-1.29	0.73	0.277	0.42-1.29	0.81	0.343	0.52-1.26
**Race**									
White	Reference			Reference			Reference		
Black	1.10	0.458	0.86-1.40	1.37	0.094	0.95-1.98	1.32	0.115	0.94-1.85
Others	0.67	0.021	0.48-0.94	0.53	0.083	0.25-1.09	0.75	0.335	0.42-1.35
**Marital status**									
Married	Reference			Reference			Reference		
Unmarried/ Loss of marriage	1.04	0.732	0.85-1.27	0.99	0.958	0.73-1.35	1.13	0.353	0.87-1.47
**Grade**									
I	Reference			Reference			Reference		
II	2.27	0.005	1.28-4.02	1.56	0.214	0.77-3.15	1.89	0.073	0.94-3.80
III-IV	3.66	<0.001	2.06-6.51	2.73	0.006	1.33-5.59	3.41	0.001	1.65-7.07
**T Stage**									
0-1	Reference			Reference			Reference		
2	0.97	0.836	0.70-1.34	0.96	0.845	0.63-1.46	0.92	0.672	0.64-1.34
3	1.49	0.021	1.06-2.09	1.53	0.059	0.98-2.37	1.35	0.132	0.91-1.98
4	2.21	<0.001	1.54-3.15	2.42	0.003	1.35-4.32	2.58	<0.001	1.59-4.18
**N Stage**									
2	Reference			Reference			Reference		

3	1.51	<0.001	1.24-1.84	1.56	0.003	1.16-2.09	1.54	0.002	1.17-2.01
**Molecular subtype**									
Hormone receptor+/ HER 2-	Reference			Reference			Reference		
Hormone receptor+/ HER 2+	0.70	0.046	0.50-0.99	0.57	0.060	0.31-1.02	0.73	0.205	0.44-1.19
Hormone receptor-/ HER 2+	1.39	0.067	0.98-1.98	1.50	0.138	0.88-2.58	1.18	0.415	0.79-1.78
Hormone receptor-/ HER 2-	3.15	<0.001	2.45-4.04	3.17	<0.001	2.14-4.69	2.93	<0.001	2.02-4.23
**Radiotherapy**									
No/unknown	Reference			Reference			Reference		
Yes	0.69	<0.001	0.56-0.84	0.75	0.065	0.55-1.02	0.69	0.013	0.51-0.93
**Chemotherapy**									
No/unknown	Reference			Reference			Reference		
Yes	0.55	<0.001	0.42-0.70	0.76	0.288	0.45-1.26	0.65	0.008	0.47-0.89
**Surgery**									
Mastectomy	Reference			Reference			Reference		
PMbR	0.83	0.149	0.64-1.07	0.86	0.310	0.64-1.15	0.84	0.298	0.61-1.17

Abbreviation: CI, confidence interval; HER2, human epidermal growth factor receptor 2; HR, hazard ratio; PMbR, post-mastectomy breast reconstruction.

* P-values showed whether there were statistical differences among, new P-values shall be shown, when necessary, in the below.

Table S2. Clinicopathological characteristics of the original cohort, matched cohort, and weighted cohort in the sensitivity analysis.

Covariates	Original Cohort				Matched Cohort				Weighted Cohort			
	Mastectomy (%) n = 1,784	PMbR (%) n = 761	*P**	SMD	Mastectomy (%) n = 716	PMbR (%) n = 716	*P**	SMD	Mastectomy (%) n = 2,526	PMbR (%) n = 2,212	*P**	SMD
**Age at diagnosis**												
<40	120 (6.7)	134 (17.6)	<0.001	0.753	99 (13.8)	112 (15.6)	0.415	0.070	243 (9.6)	232 (10.5)	0.075	0.089
40-64	933 (52.3)	542 (71.2)			541 (75.6)	519 (72.5)			1,468 (58.1)	1,303 (58.9)		
≥65	731 (41.0)	85 (11.2)			76 (10.6)	85 (11.9)			815 (32.3)	677 (30.6)		
**Race**												
White	1176 (65.9)	593 (77.9)	<0.001	0.285	541 (75.6)	549 (76.7)	0.880	0.027	1,755 (69.5)	1,599 (72.3)	0.302	0.082
Black	333 (18.7)	107 (14.1)			112 (15.6)	106 (14.8)			438 (17.3)	378 (17.1)		
Others	275 (15.4)	61 (8.0)			63 (8.8)	61 (8.5)			334 (13.2)	236 (10.6)		
**Marital status**												
Married	893 (50.1)	500 (65.3)	<0.001	0.312	444 (62.0)	454 (63.4)	0.623	0.029	1,378 (54.5)	1,256 (56.8)	0.378	0.046
Unmarried/ Loss of marriage	891 (49.9)	264 (34.7)			272 (38.0)	262 (36.6)			1,148 (45.5)	956 (43.2)		
**Grade**												
I	160 (9.0)	82 (10.8)	<0.001	0.213	79 (11.0)	77 (10.8)	0.984	0.009	241 (9.5)	238 (10.8)	0.480	0.062

II	745 (41.8)	384 (50.5)			348 (48.6)	350 (48.9)			1,119 (44.3)	1,016 (45.9)		
III-IV	879 (49.3)	295 (38.8)			289 (40.4)	289 (40.4)			1,166 (46.2)	959 (43.3)		
**T Stage**												
0-1	236 (13.2)	128 (16.8)	<0.001	0.392	116 (16.2)	121 (16.9)	0.842	0.048	366 (14.5)	366 (16.5)	0.025	0.164
2	759 (42.5)	358 (47.0)			331 (46.2)	340 (47.5)			1,106 (43.8)	989 (44.7)		
3	495 (27.7)	239 (31.4)			227 (31.7)	219 (30.6)			725 (28.7)	677 (30.6)		
4	294 (16.5)	36 (4.7)			42 (5.9)	36 (5.0)			329 (13.0)	180 (8.1)		
**N Stage**												
2	1,099 (61.6)	513 (67.4)	0.006	0.122	486 (67.9)	472 (65.9)	0.465	0.042	1,596 (63.2)	1,431 (64.7)	0.534	0.032
3	685 (38.4)	248 (32.6)			230 (32.1)	244 (34.1)			930 (36.8)	781 (35.3)		
**Molecular subtype**												
Hormone receptor+/ HER 2-	1124 (63.0)	555 (72.9)	<0.001	0.226	521 (72.8)	516 (72.1)	0.951	0.031	1,668 (66.0)	1,549 (70.0)	0.414	0.086
Hormone receptor+/ HER 2+	258 (14.5)	93 (12.2)			87 (12.2)	88 (12.3)			346 (13.7)	274 (12.4)		

Hormone receptor-/ HER 2+	143 (8.0)	43 (5.7)			38 (5.3)	43 (6.0)			184 (7.3)	138 (6.3)		
Hormone receptor-/ HER 2-	259 (14.5)	70 (9.2)			70 (9.8)	69 (9.6)			328 (13.0)	251 (11.4)		
**Radiotherapy**												
No/unknown	573 (32.1)	215 (28.3)	0.059	0.084	199 (27.8)	208 (29.1)	0.639	0.028	779 (30.8)	654 (29.6)	0.608	0.027
Yes	1,211 (67.9)	546 (71.7)			517 (72.2)	508 (70.9)			1,747 (69.2)	1,558 (70.4)		
**Chemotherapy**												
No/unknown	380 (21.3)	52 (6.8)	<0.001	0.425	46 (6.4)	72 (7.3)	0.601	0.033	430 (17.0)	267 (12.1)	0.037	0.140
Yes	1,404 (78.7)	709 (93.2)			670 (93.6)	664 (92.7)			2,096 (83.0)	1,945 (87.9)		
**Laterality**												
Left	881 (49.4)	382 (50.2)	0.739	0.016	346 (48.3)	356 (49.7)	0.634	0.028	1,246 (49.3)	1,086 (49.1)	0.930	0.004
Right	903 (50.6)	379 (49.8)			370 (51.7)	360 (50.3)			1,280 (50.7)	1,126 (50.9)		
**Histology**												
IDC	1,338 (75.0)	579 (76.1)	0.148	0.072	555 (77.5)	551 (77.0)	0.795	0.036	1,975 (78.2)	1,702 (76.9)	0.567	0.055
ILC	396 (22.2)	162 (21.3)			145 (20.3)	145 (20.3)			467 (18.5)	450 (20.3)		

Others	50 (2.8)	20 (2.6)			16 (2.2)	20 (2.8)			84 (3.3)	61 (2.8)		
**LN examined**												
≤15	930 (52.1)	380 (49.9)	0.331	0.044	355 (49.6)	353 (49.3)	0.958	0.006	1,297 (51.3)	1,080 (48.8)	0.323	0.050
>15	854 (47.9)	381 (50.1)			361 (50.4)	363 (50.7)			1,229 (48.7)	1,132 (51.1)		

Abbreviation: HER2, human epidermal growth factor receptor 2; IDC, infiltrating ductal carcinoma; ILC, infiltrating lobular carcinoma; LN, lymph node; PMbR, post-mastectomy breast reconstruction; SMD, standardized mean difference.

* P-values showed whether there were statistical differences among, new P-values shall be shown, when necessary, in the below.

Table S3. Sensitivity analysis of multivariate analysis of breast cancer-specific survival predictors using Cox proportional hazard model.

Covariates	Original Cohort			Matched Cohort			Weighted Cohort		
	HR	*P**	95% CI	HR	*P**	95% CI	HR	*P**	95% CI
**Age at diagnosis**									
<40	Reference			Reference			Reference		
40-64	0.67	0.018	0.48-0.93	0.63	0.019	0.43-0.93	0.67	0.041	0.45-0.98
≥65	0.86	0.411	0.60-1.23	0.60	0.102	0.32-1.11	0.79	0.300	0.50-1.23
**Race**									
White	Reference			Reference			Reference		
Black	1.11	0.436	0.86-1.42	1.12	0.580	0.74-1.70	1.28	0.150	0.91-1.79
Others	0.69	0.032	0.49-0.97	0.89	0.724	0.47-1.69	0.69	0.127	0.42-1.11
**Marital status**									
Married	Reference			Reference			Reference		
Unmarried/ Loss of marriage	1.01	0.919	0.82-1.25	1.13	0.475	0.81-1.57	1.08	0.550	0.84-1.40
**Grade**									
I	Reference			Reference			Reference		
II	2.50	0.004	1.34-4.66	1.78	0.156	0.80-3.96	2.30	0.031	1.08-4.91
III-IV	4.32	<0.001	2.29-8.12	4.10	0.001	1.82-9.23	4.38	<0.001	2.01-9.55
**T Stage**									
0-1	Reference			Reference			Reference		
2	1.03	0.859	0.73-1.46	0.70	0.224	0.40-1.24	1.12	0.571	0.76-1.64
3	1.58	0.012	1.11-2.27	1.21	0.544	0.66-2.20	1.60	0.025	1.06-2.43
4	2.17	<0.001	1.49-3.17	2.78	<0.001	1.83-4.22	2.50	<0.001	1.58-3.95
**N Stage**									

2	Reference			Reference			Reference		
3	1.59	<0.001	1.29-1.96	1.41	0.041	1.01-1.96	1.61	<0.001	1.23-2.11
**Molecular subtype**									
Hormone receptor+/ HER 2-	Reference			Reference			Reference		
Hormone receptor+/ HER 2+	0.71	0.056	0.49-1.01	0.70	0.224	0.40-1.24	0.73	0.159	0.46-1.13
Hormone receptor-/ HER 2+	1.32	0.140	0.91-1.90	1.21	0.544	0.66-2.20	1.13	0.581	0.74-1.72
Hormone receptor-/ HER 2-	3.09	<0.001	2.39-3.99	2.78	<0.001	1.83-4.22	2.98	<0.001	2.12-4.18
**Radiotherapy**									
No/unknown	Reference			Reference			Reference		
Yes	0.69	0.001	0.56-0.86	0.76	0.112	0.54-1.07	0.72	0.023	0.54-0.96
**Chemotherapy**									
No/unknown	Reference			Reference			Reference		
Yes	0.55	0.001	0.43-0.72	1.08	0.818	0.56-2.1	0.65	<0.001	0.47-0.88
**Surgery**									
Mastectomy	Reference			Reference			Reference		
PMbR	0.62	0.131	0.46-1.02	0.67	0.115	0.49-1.02	0.62	0.137	0.54-1.08
**Laterality**									
Left	Reference			Reference			Reference		
Right	1.09	0.377	0.90-1.34	1.25	0.169	0.91-1.71	1.18	0.179	0.93-1.50

**Histology**									
IDC	Reference			Reference			Reference		
ILC	1.05	0.768	0.76-1.44	1.12	0.658	0.68-1.82	1.04	0.850	0.72-1.50
Others	0.98	0.952	0.58-1.67	1.06	0.904	0.39-2.93	1.39	0.416	0.63-3.09
**LN examined**									
≤15	Reference			Reference			Reference		
>15	0.83	0.075	0.67-1.02	0.82	0.213	0.59-1.12	0.89	0.381	0.68-1.16

Abbreviation: CI, confidence interval; HER2, human epidermal growth factor receptor 2; HR, hazard ration; IDC, infiltrating ductal carcinoma; ILC, infiltrating lobular carcinoma; LN, lymph node; PMbR, post-mastectomy breast reconstruction.

* *P*-values showed whether there were statistical differences among, new P-values shall be shown, when necessary, in the below.
